# Bone marrow-derived fibroblast growth factor-2 induces glial cell proliferation in the regenerating peripheral nervous system

**DOI:** 10.1186/1750-1326-7-34

**Published:** 2012-07-13

**Authors:** Victor Tulio Ribeiro-Resende, Alvaro Carrier-Ruiz, Robertha M R Lemes, Ricardo A M Reis, Rosalia Mendez-Otero

**Affiliations:** 1 Laboratório de Neurobiologia Celular e Molecular, Programa de Terapia Celular e Bioengenharia, Instituto de Biofísica Carlos Chagas Filho, UFRJ, Centro de Ciências da Saúde, Bl. G, Cidade Universitária, 21949-900, Rio de Janeiro, Brazil; 2 Instituto Nacional de Ciência e Tecnologia de Biologia Estrutural e Bioimagem - INBEB, Universidade Federal do Rio de Janeiro, Rio de Janeiro, Brazil; 3 Laboratório de Microbiologia Celular, Pavilhão de Hanseníase, Instituto Oswaldo Cruz, Fundação Oswaldo Cruz, Rio de Janeiro, 21045-900, Brazil; 4 Laboratório de Neuroquímica, Programa de Neurobiologia, Instituto de Biofísica Carlos Chagas Filho, UFRJ, Centro de Ciências da Saúde, Bl. C, Cidade Universitária, 21949-900, Rio de Janeiro, Brazil

**Keywords:** Regeneration, Sciatic nerve, Dorsal root ganglia, Spinal cord, FGF-2, Schwann cell, Bone marrow derived cells, Proliferation

## Abstract

**Background:**

Among the essential biological roles of bone marrow-derived cells, secretion of many soluble factors is included and these small molecules can act upon specific receptors present in many tissues including the nervous system. Some of the released molecules can induce proliferation of Schwann cells (SC), satellite cells and lumbar spinal cord astrocytes during early steps of regeneration in a rat model of sciatic nerve transection. These are the major glial cell types that support neuronal survival and axonal growth following peripheral nerve injury. Fibroblast growth factor-2 (FGF-2) is the main mitogenic factor for SCs and is released in large amounts by bone marrow-derived cells, as well as by growing axons and endoneurial fibroblasts during development and regeneration of the peripheral nervous system (PNS).

**Results:**

Here we show that bone marrow-derived cell treatment induce an increase in the expression of FGF-2 in the sciatic nerve, dorsal root ganglia and the dorsolateral (DL) region of the lumbar spinal cord (LSC) in a model of sciatic nerve transection and connection into a hollow tube. SCs in culture in the presence of bone marrow derived conditioned media (CM) resulted in increased proliferation and migration. This effect was reduced when FGF-2 was neutralized by pretreating BMMC or CM with a specific antibody. The increased expression of FGF-2 was validated by RT-PCR and immunocytochemistry in co-cultures of bone marrow derived cells with sciatic nerve explants and regenerating nerve tissue respectivelly.

**Conclusion:**

We conclude that FGF-2 secreted by BMMC strongly increases early glial proliferation, which can potentially improve PNS regeneration.

## Background

Although peripheral nerves have the ability to regenerate their axons after total transection, full functionality is usually not recovered. Molecular and cellular events that occur during early steps of nerve regeneration include axonal fragmentation and phagocytosis by invading macrophages [1]. Schwann cells (SCs) sort axons by inserting cytoplasmic protrusions into axonal bundles and ensheathing them [2]. In the absence of contact axons, SCs assume a non-myelinating phenotype and proliferate, migrate and form structures called bands of Büngner [2-4]. Non-myelinating SCs also behave similarly to support axons to grow toward the original target tissue. This sequence of events following axonal injury is known as Wallerian degeneration [1]. SCs and satellite cells that ensheathe sensory neurons in the dorsal root ganglia (DRG) proliferate and release soluble factors when peripheral somatic nerves are injured [5]. This increases the number of satellite cells that support neuronal survival during PNS regeneration [6-9]. Basic fibroblast growth factor (FGF-2) has been described as a trophic factor that induces glial cell proliferation by binding to the FGF-2 receptor type 1/2 (FGFR 1/2) during both development and regeneration of PNS [10]. Overexpression of FGF-2 at a sciatic nerve lesion site in genetically modified adult mice increased SCs proliferation and reduced SC myelinating phenotype [11]. In addition, it has been shown that axotomy-induced loss of sensory neurons resulted in increased neuronal apoptosis and reduced neuritogenesis *in vitro* in the absence of FGFR type 3 (FGFR-3) compared to the wild-type mice [12]. Application of SCs overexpressing the 21/23-kDa isoforms of FGF-2 into long gaps (> 1 cm) of transected sciatic nerve resulted in higher numbers of regenerated axonal fibers and myelinated fibers [13]. Moreover, SCs overexpressing FGF-2 combined with embryonic tissue containing dopaminergic neurons and grafted into the striatum of a mouse model of Parkinson’s disease led to functional recovery [14].

It was also recently demonstrated an increase in the expression of FGF-2 in axons of retinal ganglion cells treated with bone marrow mononuclear cells in a model of optic nerve crush [15] and this increase was correlated with a larger number of regenerating axons [15]. In other tissues such as heart it has been shown that soluble factors derived from bone marrow derived mesenchymal cells rescue cardiomiocytes from necrosis *in vitro* and were able to promote recovery of basic parameters of cardiac function *in vivo*[16]. We have previously observed that trophic activity stimulated by bone marrow derived cells strongly increases proliferation of SCs, satellite cells, and astrocytes surrounding lumbar spinal cord motoneurons [5]. Here, we tested the possibility that bone marrow cells act by delivering FGF-2 to the gap between the proximal and distal stumps of transected sciatic nerve. Our data show that the increase in cell proliferation induced by bone marrow derived cells can be blocked by the administration of an FGF-2 neutralizing antibody both *in vivo* and *in vitro*. Using RT-PCR, we observed that the levels of FGF-2 mRNA expressed by bone marrow derived mesenchymal cells (MSC) increase when these cells are co-cultured with sciatic nerve fragments. In addition, the presence of bone marrow derived cells in the injury site induces an increase in the expression of FGF-2 in SCs, DRG satellite cells and lumbar spinal astrocytes when compared with the control untreated animals. We suggest that the both the FGF-2 released by the bone marrow derived cells and the increased expression in the resident cells of the nervous system results in an increase in glial cells proliferation and may contribute to an improvement in regeneration.

## Materials and methods

### Animals

We used 3-month-old male Lister Hooded rats (n = 25) bred at our institution’s rodent facility and housed with free access to food and water. All experiments were performed following the National Institute of Health Guidelines for the Care and Use of Laboratory Animals and approved by the Committee for the Use of Experimental Animals from the Universidade Federal do Rio de Janeiro (CEUA protocol # 064).

To obtain bone marrow cells, rats were deeply anesthetized with ether and sacrificed by cervical dislocation. The tibia and femur were removed and cleaned of muscles, and the epiphyses were cut. Bone marrow was flushed from the bones using 15 mL of DMEM F-12 (Dulbecco’s Modified Eagle Medium), and the collected cells were gently dissociated with a Pasteur pipette. The mononuclear fraction was separated using Histopaque 1083, (Sigma-Aldrich, St. Louis, MO, USA), after centrifugation at 260 × *g* for 25 min at room temperature. The mononuclear fraction layer was carefully removed, and the cells were washed 3 times with DMEM F-12. After the last wash, cells were counted and tested for viability with trypan blue (Invitrogen, Carlsbad, CA, USA).

### Surgical procedures

**Figure 1:**
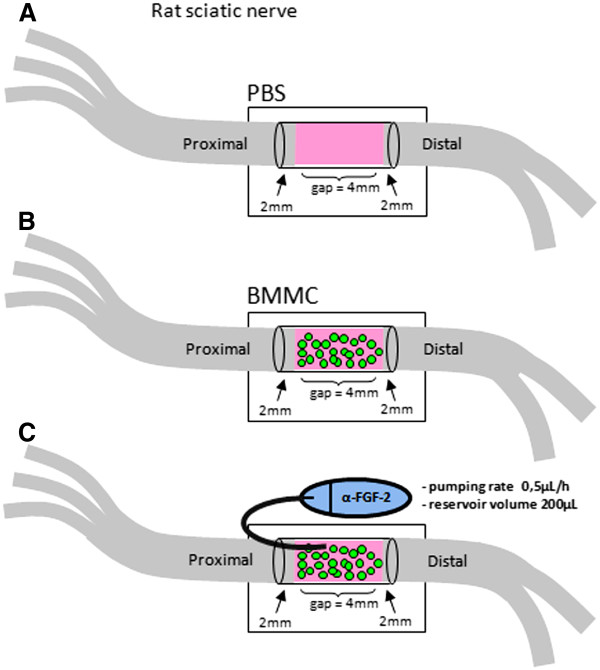
**Illustration of *in vivo *experimental model of rat sciatic nerve. A-C**: Full transection of sciatic nerve followed by connection of proximal and distal stumps inside a hollow tube of silicon and treatment with PBS (**A**), bone marrow mononuclear cells (BMMC) (**B**), or BMMC + neutralizing FGF-2 antibody delivered inside the gap by a cannula coupled to an osmotic mini-pump (**C**).

**Figure 2:**
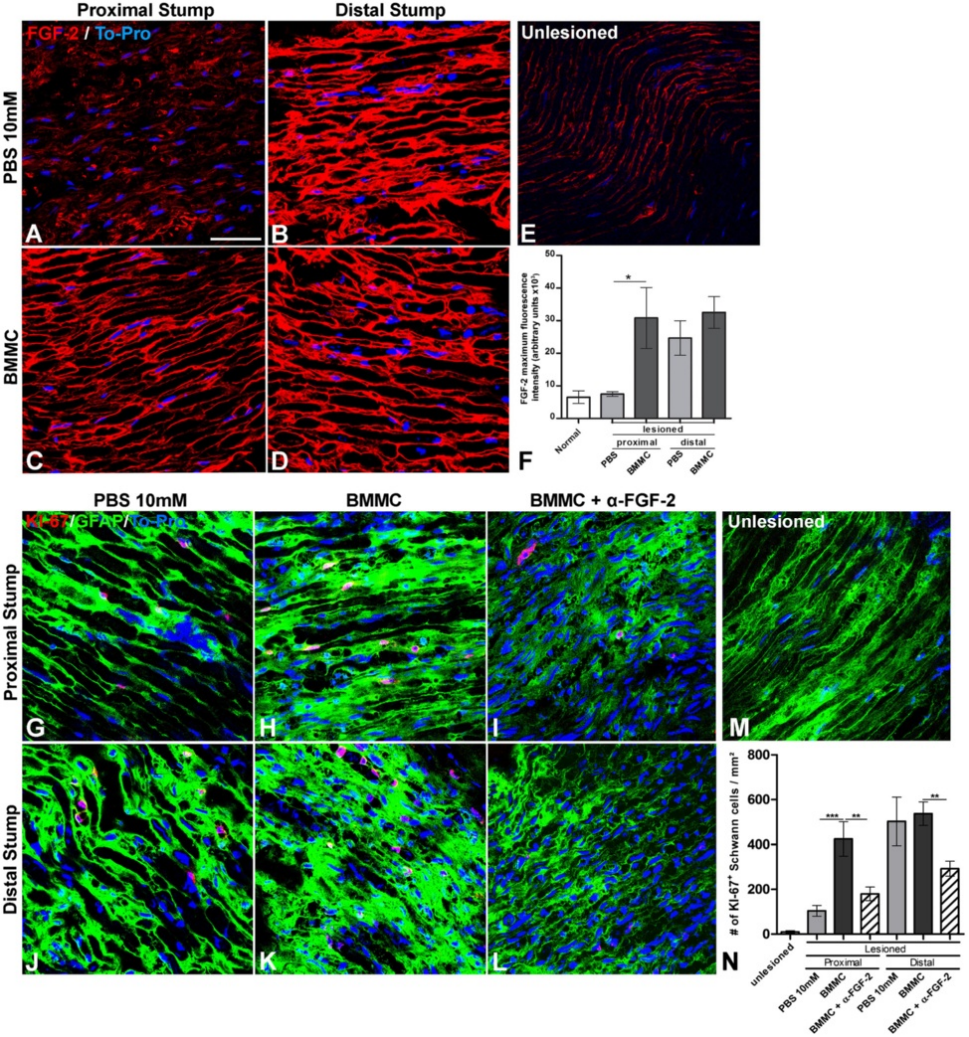
**Increased expression of FGF-2 is time correlated with increased Schwann cell proliferation in the sciatic nerve after lesion and BMMC treatment. A**-**E**: Optical sections of sciatic nerve by confocal microscopy taken 10 days after total transection immunolabeled for FGF-2 and treated with PBS (**A**, proximal stump and **B**, distal stump) or with BMMC (**C**, proximal stump and **D**, distal stump). An intact nerve is showed in **E**. Cell nuclei were counterstained with TO-PRO. **F**: Histogram of FGF-2 expression in sciatic nerve tissue comparing different experimental groups. Statistics: *p < 0.0001* ANOVA. **G**-**M**: Optical sections of sciatic nerve by confocal microscopy taken 10 days after total transection double immunolabeled for KI-67 (red) and GFAP (green) treated with PBS (**G**, proximal stump and **J**, distal stump), with BMMC (**H**, proximal stump and **K**, distal stump) or BMMC + neutralizing antibody for FGF-2 (**I**, proximal stump and **L**, distal stump) delivered by osmotic mini-pumps. Uninjured nerve is showed in **M**. **N**: Histogram of the number of KI-67+ SCs in the nerve tissue under the different experimental conditions. Statistics: *p* < 0.001 ANOVA. Scale bar: **A**-**E**; **G**-**M** = 50 μm.

Total transection and connection of the sciatic nerve (SN) was performed under anesthesia with xylazine chloride (5 g/Kg Rumpum 0.5%, Bayer, São Paulo, Brazil) and ketamine chloride (50 g/Kg Vetaset 5%, Fort Dodge Laboratories, São Paulo, Brazil). The right sciatic nerves were exposed and sectioned at the mid thigh level. Distal and proximal stumps were re-connected inside an 8-mm polyethylene tube, leaving a gap of 4 mm between both stumps inside the tube. One group of rats received 1.2 × 107 cells in 15 μL of a Matrigel solution (30% Matrigel in 10 mM phosphate buffer (PBS), Collaborative Biomedical Products, Bedford, MA, USA) (BMMC group; n = 6; Figure 1B). Cell injections were performed with a 10-μL microsyringe immediately after the surgery and reconnection (Hamilton, Reno, NV, USA). The control group received only the matrigel solution (PBS group; n = 5; Figure 1A). For the experiments with the neutralizing antibody an osmotic pump system (ALZET mini pumps, Cupertino, CA, USA) was used to deliver the neutralizing FGF-2 antibody to the tube. This group (n = 4, Figure 2C) received first the same number of cells followed immediately by a plugged needle on top of the tube that delivered antibody continuously into the gap containing BMMC. Osmotic pumps were filled up with 200 μL of solution containing 100 μg/mL of neutralizing mouse monoclonal α-FGF-2 (Santa Cruz Biotechnology, Santa Cruz, CA, USA). The delivery rate was 0.5 μL/h. After recovery from anesthesia, the animals were returned to the animal facility and kept with food and water *ad libitum* for 10 days.

### Histology

Rats were deeply anesthetized in the CO2 chamber and perfused with paraformaldehyde 4% (PF 4% in 0.1 M PBS, pH 7.4) to examine the role of FGF2 on glial cell proliferation ten days after surgical procedures. The sciatic nerve (SN), DRG (L5) and lumbar spinal cord (LSC) were removed and kept in a 30% sucrose solution in 0.1 M PBS, pH 7.4 for 48 h. Tissue samples were mounted in optimal cutting temperature (OCT) compound (Sakura Fine Technologies, Zoeterwoude, Netherlands). Frozen longitudinal sections (16 μm) of SN and DRG and transverse sections of LSC (16 μm) were cut on a cryostat (Leica CM 1850, Wetzlar, Germany) and mounted directly onto gelatin pre-coated slides. DRG and LSC sample sections were stained with neutral red (1% neutral red in 0.1 M acetate buffer, pH 4.8). These sections were dehydrated, mounted with Enthelan (Merck, Rio de Janeiro, Brazil) and analyzed on an Axiovert 135 microscope equipped with an Axiocam (Zeiss, Aktiengesellschaft, Germany). Another group of slices was stored at −20°C for immunofluorescence procedures.

### Culture of bone marrow-derived mesenchymal cells

After harvest (described above), bone marrow cells were plated in DMEM F-12 with 10% fetal bovine serum (FBS, Invitrogen), penicillin and streptomycin (10,000 units/mL and 10 mg/mL respectively, Sigma), fungizone (10 mg/mL, Sigma), glutamine (100 mg/mL, Invitrogen) and sucrose (0.15%, Sigma). Cells (2 × 105) were added to 10-cm dishes (Corning, Corning, NY, USA) and kept in the incubator with 5% CO2 overnight at 37°C. Non-adherent cells were removed and adhered cells were supplemented with fresh DMEM F-12 + 10% fetal bovine serum (FBS) after 24 h in culture. Confluent cells in passage number 3 were kept in culture for 72 h with standard media (DMEM F-12 + 10% FBS). Then, BMMC conditioned medium (BMMC-CM) was collected, centrifuged at 260 × *g* for 10 min, filtered with a 0.22 μm filter and frozen at −20°C.

### Schwann cells and *in vitro* proliferation assay

The ST-8814 human lineage of SCs was cultured with DMEM F12 + 10% FBS, until 70% confluent in 24-well culture dish (Corning). Cells were then re-plated and incubated with CM diluted 1:1 with standard medium after 3 passages. Another group of cells was incubated with CM + neutralizing mAb anti-FGF-2 (1:1000). Recombinant human FGF-2 (rhFGF-2, Invitrogen) was also added as a positive control, as well as the same recombinant protein in the presence of the neutralizing mAb as a control for neutralization. The control group of cells was kept with DMEM F-12 + 10% FBS. All groups were then incubated for 72 h in 5% CO2 at 37°C. After that, cells were washed once with 10 mM PBS, pH 7.4 and fixed with paraformaldehyde 4% (PF 4%) in PBS, pH 7.4. To investigate the proliferative effect of CM on cultured SCs, we performed immunostaining for KI-67, a marker of proliferation. Fixed Schwann cells were washed 3 times for 5 min each with 10 mM PBS, pH 7.4, followed by incubation with 5% normal goat serum (NGS) for 30 min and KI-67 IgG rabbit polyclonal antibody (1:200, Abcam, Cambridge, MA, USA) overnight. Afterwards, cells were washed again 3 times with 10 mM PBS for 5 min and mounted with coverslips containing one drop of Vectashield with 4’, 6’-diamidino-2-phenylindole (DAPI, Vector, Burlingame, CA, USA) for nuclei counterstaining. KI-67+ Schwann cells were counted and compared among the different experimental conditions.

### DRG explants and *in vitro* neurite growth assay

DRG explant cultures were obtained from E16 rat embryos. Pregnant rats were sacrificed by cervical dislocation, and the embryos were removed immediately. DRGs were dissected and incubated in DMEM F-12 with 50 ng/mL NGF (Invitrogen) for 1 h at 36°C and 5% CO2 before plating on coverslips pre-coated with 100 μg/mL of poly-L-lysine (Sigma) and 50 μg/mL of laminin (Invitrogen). DRGs explants were plated in 6 experimental groups. The control group was cultured with DMEM F-12 (n = 5). A second group was kept with CM diluted 1:1 with standard medium (DMEM F-12 + 10% FBS, n = 5). A third group had CM + neutralizing FGF-2 antibody (2.5 μg/mL, Abcam). A fourth group had recombinant human FGF-2 protein (rhFGF-2, 20 ng/mL, Invitrogen) added to the standard medium. The fifth group received rhFGF-2 + neutralizing antibody for FGF-2 (same concentration as described above) and the last group received rhFGF-2 and rhNGF (20 ng/mL both, Invitrogen) . DRG explants were incubated for 48 h and then washed once with 10 mM PBS, fixed with PF 4% and double immunostained with antibodies against Tuj-1 (1:500, Covance, Princeton, NJ, USA) and glial fibrillary acidic protein (GFAP, 1:400, DAKO, São Paulo, Brazil) following the same procedures as described above for cultured SCs. Cell nuclei were counterstained with DAPI-containing Vectashield. Neurite growth was assessed by confocal microscopy (LSM 510 Meta, Zeiss). The number of neurites from the DRG neurons was counted and compared among all experimental conditions.

### Dissociated DRG neurons *in vitro*

DRG explants were obtained from E14 rat embryos as described above. DRGs were dissected and incubated in DMEM F-12 with 50 ng/mL NGF (Invitrogen) for 1 h at 36°C in 5% CO2. Ganglia were cleaned and incubated at 37°C with 0.05% trypsin for 10 min in Ca2+ and Mg2+ Free Hanks’ solution (CMF). After centrifugation and removal of the trypsin solution, the ganglia were washed with 10 mL of DMEM and 10% FBS, and triturated with a fire-polished Pasteur pipette. Neurons and glial cells were plated at a low density on poly-L*-*lysine- (10 μg/ml) and laminin- (20 μg/ml) coated 4-well dishes (Nunc Inc., Rochester, NY, USA) [17]. Culture conditions were the same as described above for DRG explants. The neurons were incubated at 37°C in a humidified 5% CO2 incubator for 48 h. At least three independent counts were repeated for each experimental paradigm. Neurite growth was assessed with confocal microscopy after double immunostaining for Tuj-1 and GFAP.

### Sciatic nerve explants and SC migration assay

**Figure 3:**
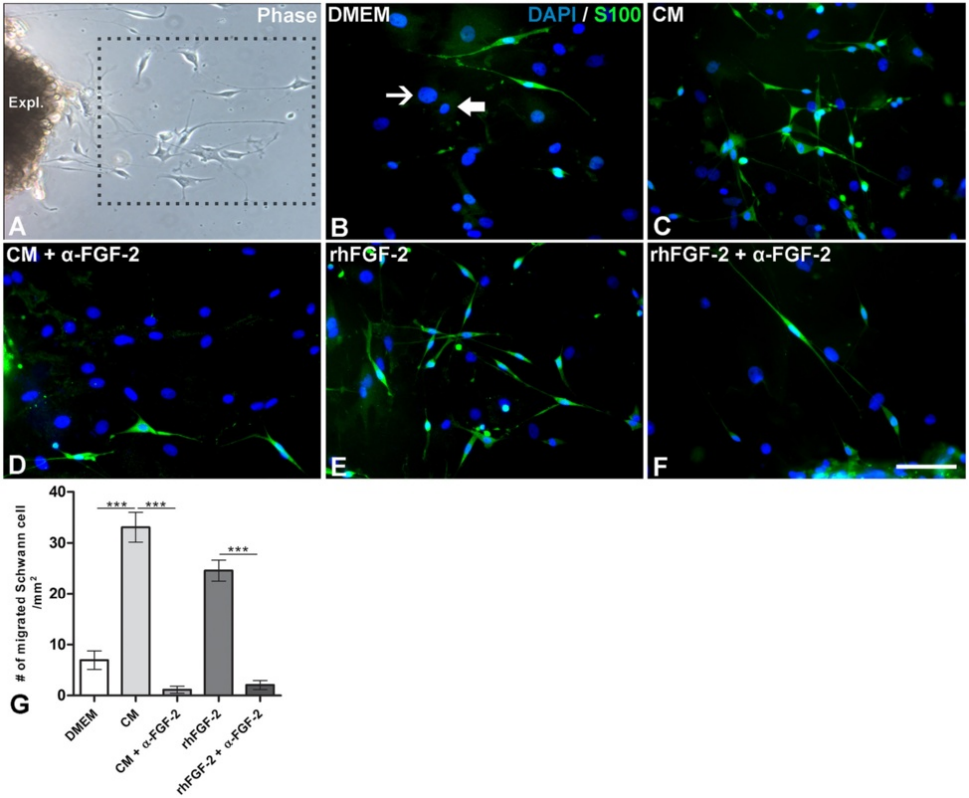
**FGF-2 secreted by BMMC increases SC migration from sciatic nerve explants. A-F:** Crushed sciatic nerve explants from distal nerve stump kept for ten days in culture under specific experimental conditions. **A:** Phase contrast image showing one explant with cells migrating from the tissue. **B-F:** Epifluorescent microscopy of migrating SCs immunolabeled for S-100β (green) and incubated with control medium (**A**), BMMC-CM (**B**), BMMC-CM+ neutralizing FGF-2 (**C**), rhFGF-2 added to the control medium (**D**) or rhFGF-2 + neutralizing FGF-2 (**E**). Cell nuclei were counterstained with DAPI. **F:** Quantitative analysis of the number of S-100β migrated SCs from sciatic nerve explants after incubation under different experimental conditions. Statistics: p<0.0001 ANOVA. Non-Schwann cells are indicated by thick or thin arrows in **B.** Scale bar: **A-F**= 50 µm.

Two rats had both sciatic nerves crushed at the mid-thigh level under anesthesia as described above. After 24 h these animals were sacrificed by cervical contusion, and the nerves were carefully removed. The aim of this procedure was to activate Schwann cells at distal stump after lesion since axons were disrupted. Then, the epineurium tissue from the distal nerve stumps was removed under low magnification microscopy, and small pieces of nerve (explants) were removed with ophthalmic scissors. One explant per well was placed in a 24-well culture dish containing coverslips pre-coated with 100 μg/mL of poly-D-lysine (Sigma) and 50 μg/mL of laminin (Invitrogen). Explants were kept at 37°C, 5% CO2 for 5 days with DMEM F-12 + 20% FBS, and 5 days more under the same experimental conditions as described above for cultured Schwann cells. Then, the cultures were fixed with 4% PF for 15 min, washed 3 times for 5 min each with 10 mM PBS, pH 7.4 and immunostained for S100-β (1:400, rabbit polyclonal, DAKO). Anti-rabbit IgG Alexa 555 (Invitrogen, 1:800) was applied as secondary antibody. Cell nuclei were counterstained with DAPI-containing Vectashield. Fluorescence was imaged by an Apotome system (Zeiss, Germany) coupled to a microscope (Axiovert 200 M, Zeiss, Germany) and captured by a camera (Axiocam, Zeiss). The number of S100-β + Schwann cells that migrated from the nerve explants were counted in a fixed area represented by the dashed square in Figure 3A and compared among all experimental conditions. The total number of cells that migrated (DAPI+) from explants was also accounted following the same parameters.slices by confocal microscopy of longitudinal sections of DRG double immunolabeled for KI-67 (red), GFAP (green) and counterstained with TO-PRO (blue) ten days after sciatic nerve lesion treated with PBS (**G**), BMMC (**H**), BMMC + neutralizing FGF-2 antibody (**I**) delivered by osmotic mimi-pumps or uninjured sciatic nerve (**F**). **J**: Quantification of the number of KI-67+ cells per mm2 of DRG tissue comparing the experimental groups. Statistics: *p <* 0.0001 ANOVA. Scale bar: **A** = 200 μm; **B-D**, **F**-**I** = 50 μm.

### Immunofluorescence procedures

For double immunostaining with KI-67 and GFAP, frozen sections were equilibrated to room temperature (RT) inside a humid chamber. The slides were then placed in a 4% PF wet chamber for 30 min to promote adhesion of the sections to the slides. Next, slides were washed twice for 5 min each with 10 mM PBS, pH 7.4 at RT prior to 30 min incubation in 0.01 M citrate buffer, pH 6.0 at 95°C. Then, slides were washed three times with 10 mM PBS, pH 7.4 + 0.3% Triton X-100. After these procedures, slides were incubated with 5% normal goat serum (NGS, Invitrogen) in wash solution for 1 h at RT. Incubation with the primary antibodies KI-67 rabbit monoclonal (1:50, Abcam) and GFAP mouse monoclonal (1:400, Dako) was performed overnight at 4°C followed by 3 washes with 10 mM PBS + 0.3% Triton X-100 (5 min each) and then sections were incubated with appropriate secondary antibodies Alexa Fluor 488-conjugated goat anti-mouse (1:400, Invitrogen) and Cy3 goat anti-rabbit (1:400, Jackson Laboratories, Bar Harbor, ME, USA) for 2 h at room temperature. After 3 washes, sections were mounted with Vectashield with DAPI (Vector) and analyzed with an epifluorescent microscope (Axiovert 200 M, Zeiss) or confocal microscope (LSM 510 META, Zeiss). Neurons co-cultured with SCs were also fixed with paraformaldehyde 4%, washed 3 times with 10 mM PBS + 0.1% triton, incubated with 5% NGS for 1 h followed by incubation with monoclonal anti-mouse Tuj-1 (1:500, Covance) for 2 h. Cells were again washed 3 times with 10 mM PBS and incubated with anti-mouse Cy3 (1:400, Jackson laboratories). Again, neurite outgrowth was assessed with confocal microscopy (LSM 510, Zeiss).

### Western blotting

For western blotting assay, SCs were incubated under different experimental conditions as described above, with medium containing 0.1% FBS for 22 h followed by 2 h of culture serum withdrawal. After, SCs were washed twice with cold 10 mM PBS, pH 7.4 containing Ca2+. RIPA buffer complemented with 1 mM sodium orthovanadate and 1 mM sodium fluoride was added to the plates to lyse cells, and the mixture was incubated for 20 min at 4°C, followed by DNA shearing. Samples were then treated with sample buffer and resolved by 15% SDS-PAGE gels, and then transferred to nitrocellulose membranes. After blocking with TBS + 3% BSA for 2 h at room temperature, membranes were incubated with anti-phospho Erk 1/2 (1:2000, rabbit polyclonal, Cell Signaling, Danvers, MA, USA), anti-phospho Akt 1/2/3 (1:1000, rabbit polyclonal, Santa Cruz Biotechnology, Santa Cruz, CA, US), or anti-Erk (1:1000, rabbit polyclonal, Santa Cruz Biotechnology, Santa Cruz, CA, USA) and anti-Akt 1/2/3 (1:1000, rabbit polyclonal Santa Cruz Biotechnology, Santa Cruz, CA, USA) for 2 h at room temperature. Protein bands were visualized by incubation with goat anti-rabbit horseradish peroxidase-conjugated antibody (1:40.000, Bio-Rad Laboratories, CA, USA) and ECL Western blot analysis system (Amersham Pharmacia Biotech, Piscataway, NJ, USA). Images were scanned and intensity analysis was carried out using Image J software.

### Reverse transcription polymerase chain reaction (RT-PCR)

Total RNA was extracted from BMMC or MSC incubated with or without sciatic nerve fragments using TRIzol reagent (Invitrogen). Total RNA (2 μg per sample) was treated with amplification-grade DNAse I (Invitrogen) and reverse-transcribed with Superscript II Reverse Transcriptase (Invitrogen) and OligodT18 (IDT, Coralville, IA, USA). PCR reactions were performed with Platinum® Taq DNA Polymerase (Invitrogen). RNA extraction, cDNA synthesis, and PCR reactions were performed according to the manufacturers’ instructions. FGF-2 and GAPDH were amplified using a melting temperature of 60°C. PCR products were analyzed by electrophoresis on 1.5% agarose gels stained with ethidium bromide. GAPDH was used as an internal amplification control. The following primer sequences were used: FGF-2 5-AGGAAGATGGACGGCTGCTG (forward) and 5-GCCCAGTTCGTTTCAGTGCC (reverse); GAPDH 5-ATCAAGAAGGTGGTGAAGCAGG (forward) and 5-AGGTGGAAGAGTGGGAGTTGCT (reverse).

### Quantitative analyses and statistics

KI-67/GFAP + cells were counted in optical sections obtained by confocal microscope (LSM 510 Meta, Zeiss, Germany). Longitudinal sections of SN (n = 14) and DRG L4/L5 (n = 14) and transversal sections from lumbar spinal cord (n = 14) were analyzed after immunostaining, as described above. *In vitro* neurite extension from the DRG neurons was assessed from images using Axiovision 4.3 software (Carl Zeiss, Germany). The same software was also employed to count the number of SCs and non-SCs that migrated from the sciatic nerve explants.

Statistical analyses were performed using one way analysis of variance (ANOVA) followed by a Neuman-Keuls post-test comparing all pairs of columns. All data are expressed as mean ± standard error of the mean (SEM). Symbols in the histograms: * *p* < 0,01; ** *p* < 0,001 and *** *p* < 0,0001.

## Results

### Expression of FGF-2 in regenerating nerves and increased glial proliferation after BMMC treatment

**Figure 4:**
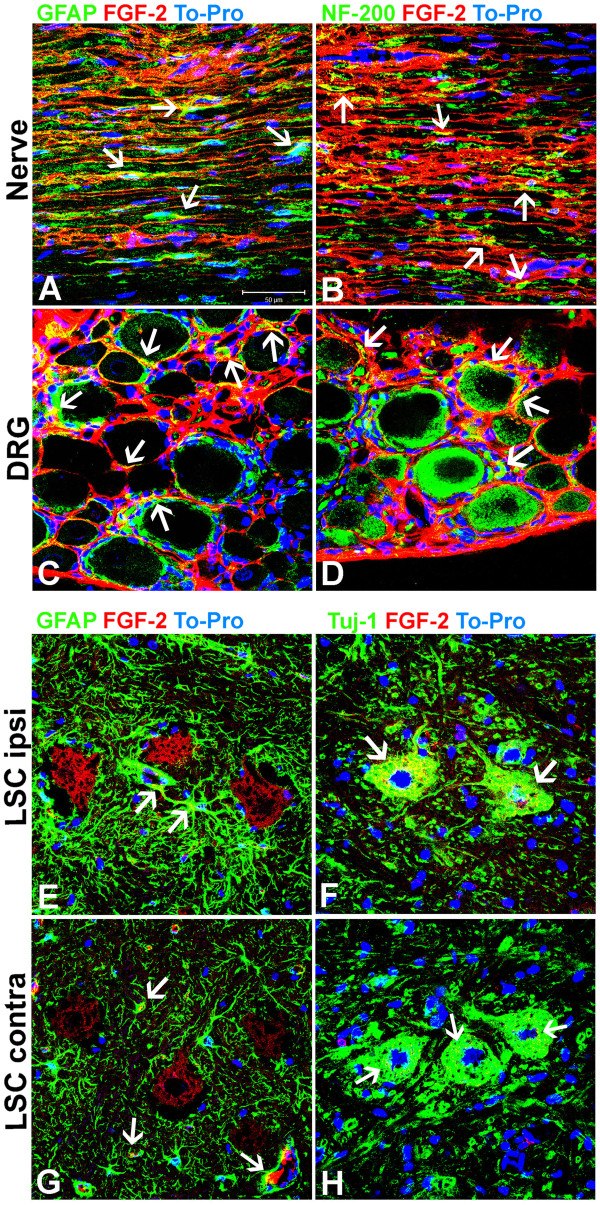
**Localization of FGF-2 in the regenerating nerve tissue, DRG and lumbar spinal cord. A-H:** Optical slices by confocal microscopy of longitudinal sections of sciatic nerve at proximal stump (**A** and **B**) or dorsal root ganglia (**C** and **D**) and transversal section of lumbar spinal cord (**E-H**) at the ipsi-lateral (**E** and **F**) or contra-lateral region (**G** and **H**). All samples were obtained from rats 10 days after treatment with BMMC. Sciatic nerve and DRG sections were double immunolabeled for GFAP/FGF-2 (A and C) and NF-200/FGF-2 (**B** and **D**). LSC sections were double immunolabeled for GFAP/FGF-2 and Tuj-1/FGF-2. White arrows indicate yellow areas of colocalisation between glial marker (GFAP) and FGF-2 and neuronal markers (NF-200/Tuj-1) and FGF-2. Scale bar: A-H = 50 βm.

Figure 1 illustrates the experimental design for injection of bone marrow derived cells (~2,5x10^7^, Figure 1B), vehicle (PBS, Figure 1A) or neutralizing anti-FGF-2 antibody (Figure 1C) into a polyethylene tube reconnecting both stumps (4 mm gap) after sciatic nerve transection of 3-month-old Lister Hooded male rats. Immunofluorescent labeling of longitudinal sections of the sciatic nerve (Figure 2A-E) shows that FGF-2 expression is increased at the proximal stump in rats that received bone marrow cells (Figure 2C) compared to the vehicle group (Figure 2A) 10 days after injury (DAI). On the other hand, FGF-2 expression had basal levels on unlesioned control rats (Figure 2E). Colocalization of FGF-2 and Erb-B2 receptor is found on the surface of SCs (Additional file 1: Figure S1). The quantitative analysis among the experimental groups is shown in Figure 2F. Since an increased expression of FGF-2 is correlated with cell proliferation in the regenerating nerve tissue, the number of KI-67/GFAP + SC nuclei was evaluated at the proximal stump. As shown, it was found to be significantly increased in the treated group as compared to theother groups (Figure 2G, H, N). This proliferation is concurrent with the increase in FGF-2 expression in the regenerating proximal stump. The expression of FGF-2 in both experimental conditions was higher at the distal stump compared to the proximal stump (Figure 2B, D, F). However, there were no differences in either FGF-2 expression or SC proliferation between BMMC and PBS groups in the distal stump (Figure 2J, K, N). The same is true for SC proliferation; the number of KI-67/GFAP + SC was increased at the proximal stump of nerves treated with cells when compared with the other groups (Figure 2H, N). Addition of the neutralizing FGF-2 antibody in rats that received cells decreased the expression levels of FGF-2 (data not shown) and also SC proliferation in the regenerating nerve tissue compared to the vehicle group in both the proximal and distal stumps (Figure 2I, L, N). Control rats with unlesioned nerves had low levels of expression of FGF-2 and KI-67 (Figure 2E, F, M, N). To address whether FGF-2 is expressed by axons, Schwann cells or both we double immunolabeled longitudinal section of cell treated sciatic nerves at the proximal stump with anti-GFAP and anti-FGF-2 antibodies (Figure 4A) or with ant-NF-200 and anti-FGF-2 antibodies (Figure 4B). We found co-localisation of FGF-2 with GFAP and also with NF-200 suggesting that FGF-2 is present in both SC and axons in the regenerating nerve tissue. 

### Increased expression of FGF-2 in the dorsal root ganglia (DRG) after cell treatment

**Figure 5:**
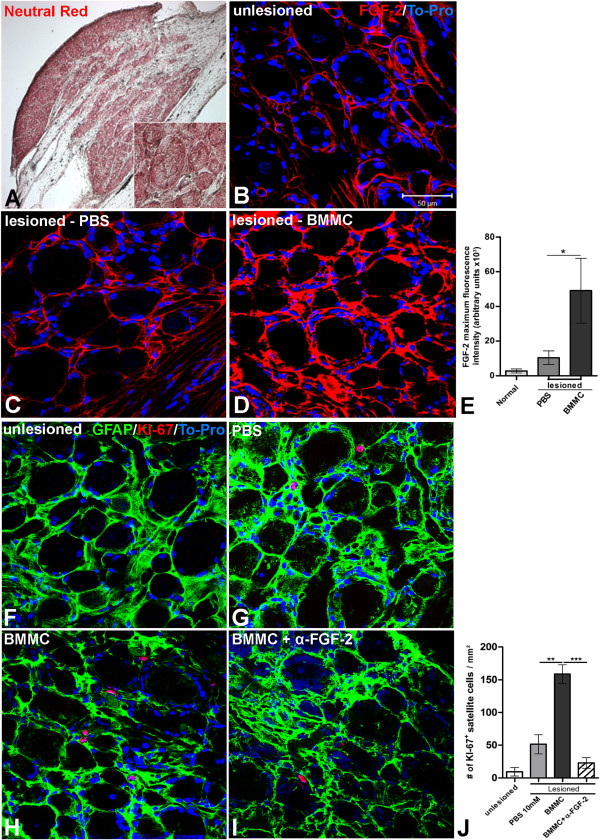
**Increased expression of FGF-2 is time correlated with increased satellite cell proliferation in the DRG after nerve lesion and BMMC treatment. A-D; F-I:** Longitudinal sections of dorsal root ganglia (DRG) of adult rats. **A:** Low magnification photomicrograph of a longitudinal section of an uninjured DRG stained with neutral red. Insert show neurons in different cell size (low, medium and big size). **B-D:** Optical sections of DRGs slices by confocal microscopy taken 10 days after total transection immunolabeled for FGF-2 (red) and counterstained with TO-PRO (blue) treated with PBS (**C**) or BMMC (**D**) or uninjured sciatic nerve (**B**). **E:** Quantification of the fluorescence intensity compared between the experimental groups cited above. Statistics: p<0,0001 ANOVA. **F-I:** Optical.

Expression of FGF-2 was investigated in DRGs with disrupted sensory fibers growing their axons toward the sciatic nerve together with axons of motoneurons present in the L5 LSC. A representative low-magnification image of longitudinal sections of L5 DRGs in a control rat stained with neutral red is shown in Figure 5A. Also, an insert is shown for large, medium and small sensory neurons. Immunofluorescently labeled cells for FGF-2 is shown at basal levels staining in PBS-treated transected nerves at ten DAI (Control group, Figure 5B, C, E). Transplantation of bone marrow-derived cells into the injury site significantly increased the expression of FGF-2 compared to the vehicle group (Figure 5D, E). Double immunolabeling for KI-67 and GFAP in serial sections indicate the basal proliferation rate of satellite cells in uninjured group (Figure 5F, J), but upon injury, a ~5-fold increase is observed in the number of KI-67+ satellite cells (Figure 5H, J). Cells treatment increased the number of KI-67+ satellite cells 2- and 15-fold compared to the vehicle and uninjured groups, respectively (Figure 5H, J). Delivery of neutralizing FGF-2 antibody in the cell treated groupreduced the number of KI-67+ satellite cells approximately 7-fold compared to the cell-only group (Figure 5I-J). To address whether FGF-2 is expressed by DRG neurons, satellite cells or both, we immunostained longitudinal section of DRG of rats treated with cells with anti-GFAP and anti-FGF-2 antibodies (Figure 4C) or with anti-NF-200 and anti-FGF-2 antibodies (Figure 4D). We observed a strong co-localisation of FGF-2 with GFAP but almost none with NF-200 indicating that FGF-2 is mainly expressed by satellite cells but not by the neurons in the DRGs. There were few dots of co-localization of FGF-2 and NF-200. FGF-2 which are probably axons in the vicinity of the cell bodies (Figure 4D arrows). These data suggest that the observed overexpression of FGF-2 in the DRG tissue is induced by treatment with bone marrow-derived cells and subsequent increased satellite cell proliferation.

### Expression of FGF-2 and astrocyte proliferation in the LSC after cell treatment

**Figure 6:**
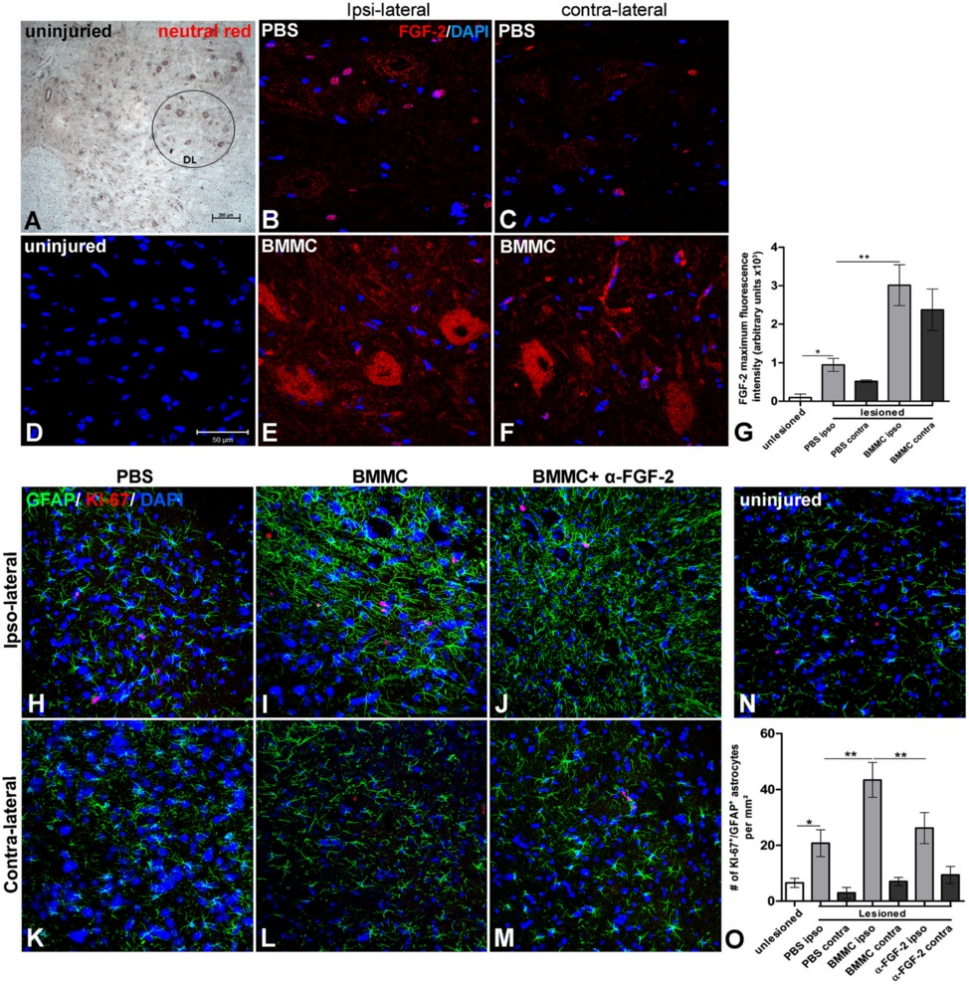
**Increased expression of FGF-2 is time correlated with increased astrocyte proliferation in the LSC after nerve lesion and treatment with BMMC. A:** Low magnification photomicrogragh of lumbar spinal cord with an uninjured sciatic nerve stained with neutral red. The DL region is circled. **B-F:** Optical sections by confocal microscopy of LSC at DL region immunolabeled for FGF-2 ten days after sciatic nerve lesion treated with PBS (**B**, ipsi-lateral and **C**, contralateral) or BMMC (**E**, ipsi-lateral and **F**, contralateral). **D:** LSC section of an uninjured rat. Cell nuclei were counterstained with TO-PRO G: histogram of FGF-2 fluorescence between experimental groups. Statistics: p<0.001 ANOVA. **H-N:** Optical slides by confocal microscopy of the DL region of LSC double immunolabeled for KI-67 (red) and GFAP (green) ten days after sciatic nerve lesion treated with PBS (**H**, ipsi-lateral and **K**, contralateral), BMMC (**I**, ipsilateral and **L**, contralateral) or BMMC+ neutralizing FGF-2 antibody (**J**, ipsilateral and **M**, contralateral) delivered by osmotic mini-pumps. **N:** LSC section of an uninjured rat. **O:** Histogram of the number of KI-67/GFAP + astrocytes at DL/LSC region per mm2. Statistics: p<0.001 ANOVA. Scale bars: **A** = 200 µm; **B-F, H-M**=50 µm.

FGF-2 is not detected in uninjured motoneuron cell bodies or axons in sciatic nerve at DL area of the anterior horn in the LSC (Figure 6A, D, G). However, FGF-2 expression by glial and neuronal cells is significantly increased at the ipsilateral, but not the contralateral, side of the LSC 10 DAI (Figure 6B, C, G). Injection of bone marrow-derived cells further enhanced FGF-2 expression of in both ipsi- and contralateral sides compared to the uninjured or vehicle (PBS) groups (Figure 6E, F, G, *p* < 0.001). Both motoneurons and glial cells showed an increase in FGF-2 following cell treatment (Figure 6E). To investigate whether this increase was also associated with an increase in astrocyte proliferation, spinal cord sections were double labeled with GFAP and KI-67. In the uninjured group, very few cells were positive for both markers (Figure 6N-O), but these numbers increased on the ipsilateral side of the PBS group 10 DAI. Expression of GFAP and KI-67 proliferating astrocytes were similar to the uninjured group on the contralateral side (Figure 6H, K, O; Additional file 2: Figure S2). The treatment of BMMCs increased the number of KI-67+ astrocytes but not GFAP reactivity at the ipsilateral side compared to the PBS group 10 DAI. Expression of both markers was similar to the uninjured group on the contralateral side (Figure 6I, L, O) (Additional file 2: Figure S2). Delivery of BMMC in the presence of neutralizing FGF-2 antibody reduced the number of KI-67+ astrocytes (from 43.43 ± 6.21 to 26.22 ± 5.57 proliferating astrocytes per mm2; *p* < 0.001). However, this condition also had an increase in GFAP reactivity (*p* < 0.01 ANOVA) compared to the vehicle group. (Figure 6J, M, O; Additional file 2: Figure S2). To address whether FGF-2 is expressed by motoneurons, astrocytes or both cell types we double labeled transversal section of LSC with an anti-GFAP and anti-FGF-2 antibodies or with anti-NF-200 and anti-FGF-2 antibodies. At the ipsi-lateral region both motoneurons and astrocytes express FGF-2 (Figure 4E and F) and similar results were observed at the contra-lateral side (Figure 4G and H). In conclusion, cell treatment increased FGF-2 expression and astrocyte proliferation on the injured size of the spinal cord in the ventral horn where the motoneuron cell bodies that form the sciatic nerve are located. Blocking FGF-2 with aneutralizing antibody reduced astrocyte proliferation, suggesting that this effect might be due to FGF-2 released by the bone marrow derived cells.

### FGF-2 neutralization reduces SC survival and proliferation *in vitro*

**Figure 7:**
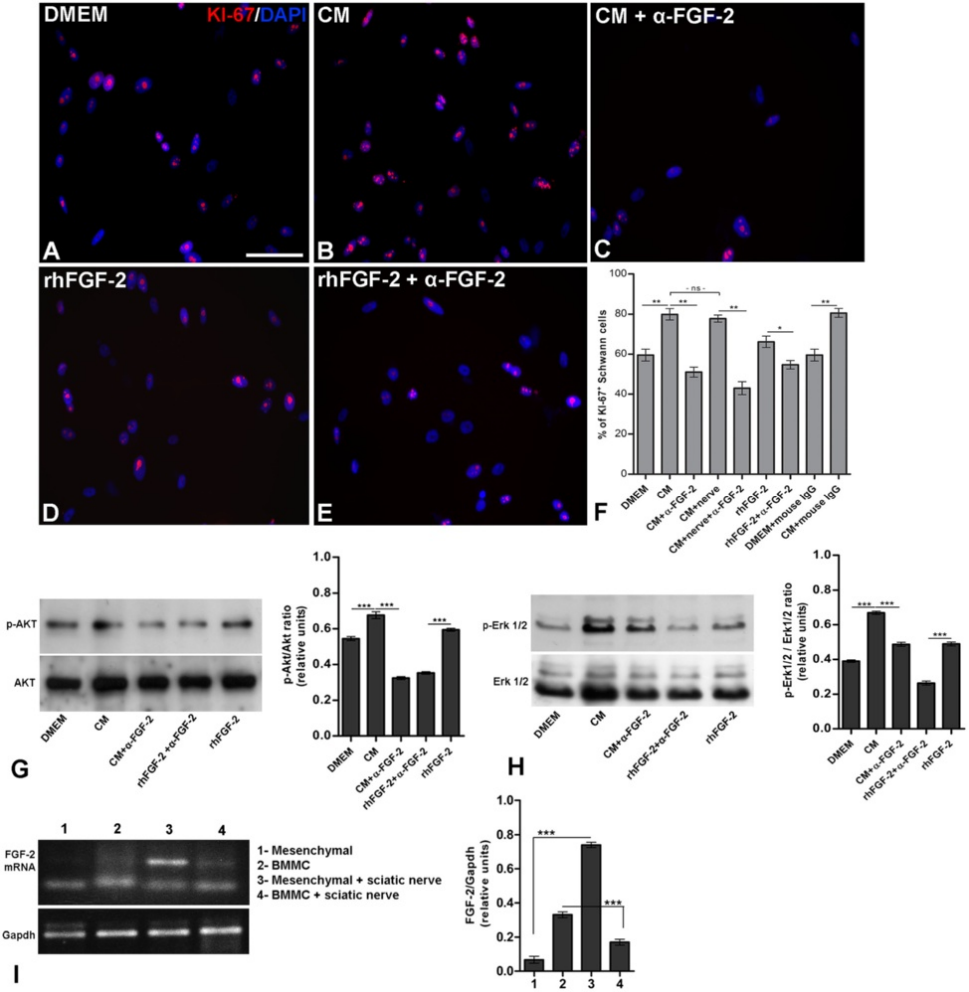
**Neutralizing FGF-2 antibody blocks SC proliferation and survival induced by BMMC-CM in vitro. A-E:** ST-8814 SCs cultured for 48 h in low density (2.00 x 104 cells/well) with DMEM F-12 + 10% FBS (**A**), CM (**B**), CM+ neutralizing FGF-2 antibody (**C**), rhFGF-2 (**D**) or rhFGF-2 + neutralizing FGF-2 antibody (**E**). Cells were immunolabeled for KI-67 and cell nuclei were counterstained with DAPI. **F:** Quantification of the percentage of KI-67+ SC per mm2. Statistics: p<0.0001 ANOVA. **G** and **H:** Western blotting and quantitative analysis of the active Akt (p-Akt, **G**) and active ERK 1/2 (p-ERK 1/2, **H**) from SCs cultured for 48 h with control medium, CM, CM+ neutralizing FGF-2, rhFGF-2 + neutralizing FGF-2 or rhFGF-2. **I:** Semi quantitative polymerase chain reaction (RT-PCR) for FGF-2 transcripts from samples of BMMC or MSC in the presence or absence of sciatic nerve pieces. Quantitative analysis showing the transcript level following the same experimental conditions. Statistics: p<0.0001 ANOVA. Scale bar: **A-E**=50 µm.

Low-density cultures of human SC line treated with CM derived from bone marrom cells had 20% more proliferating SCs after 48 h compared to the control group (Figure 7A, B and F, Additional file 3: Figure S3). Addition of the neutralizing FGF-2 antibody reduced the number of proliferating SCs in both low- and high-density cultures (30% and 15% respectively, Figure 7C and F, Additional file 3: Figure S3). Recombinant human FGF-2 protein (rhFGF-2) increased cell proliferation (Figure 7D and F), but in the presence of neutralizing FGF-2 antibody, cultures treated with rhFGF-2 had cell proliferation levels similar to that of the control (Figure 7E and F, Additional file 3: Figure S3). Conditioned medium of BMC in the presence of small pieces of injured sciatic nerve also increased SC proliferation at the same levels compared to the CM in the absence of nerve pieces (Figure 7F). Neutralizing FGF-2 antibody added to the CM of BMC in the presence of nerve pieces reduced in 38% the number of KI-67+ SC (Figure 7F). Mouse IgG was used as immunoglobulin control and did not alter SC proliferation when added to the control or CM (Figure 7F).

We analyzed the effect of neutralization of FGF-2 on the activation of phosphorylated Akt (p-Akt, Figure 7G) and extracellular signal-related kinase (p-ERK 1/2, Figure 4H), as these pathways are related to survival and proliferation of SCs. Addition of CM increased Akt and ERK phosphorylation (14% and 25% respectively, Figure 7G-H) when compared to the control group. Addition of the neutralizing FGF-2 antibody to the CM reduced the phosphorylation of Akt and ERK 1/2 compared to the CM-only group (42% and 18%,respectively). Treatment with rhFGF-2 increased Akt phosphorylation (Figure 7G, *p* < 0.01) but had no effect on ERK 1/2 (Figure 7H, *p* > 0.01), compared to the control (DMEM) group. This suggests that bone marrow-derived FGF-2 is an important trophic factor that affects the survival and proliferation of SCs. Because basal levels of phosphorylation are seen following FGF-2 neutralization, it is possible that other unidentified soluble factor(s) present in the CM also affect SC survival and proliferation.

We also analyzed the levels of FGF-2 RNA in the mononuclear fraction of BMMC and the adherent fraction of MSC cultured with or without small pieces of injured sciatic nerve (Figure 7I). Quantitative analysis showed that in MSC, the presence of the sciatic nerve increased 7-fold the amount of FGF-2 transcripts (p < 0,0001). However, BMMC cultured with small pieces of injured sciatic nerve decreased the transcript level a fold (p < 0,0001). This suggests that mesenchymal cells, which represents about 0,01% of BMMC [18], in contact with lesioned sciatic nerve overexpress FGF-2, a result that had previously been observed in the sciatic nerve tissue of rats treated with BMMC.

### Bone marrow-derived FGF-2 stimulates SC migration after nerve lesion

SCs in the presence of degenerating axons after peripheral nerve injury proliferate and migrate to form a permissive microenvironment for axonal regeneration [1]. We tested the potential of CM to induce SC migration in rat sciatic nerve explants cultured for 24 h after nerve crush. Migrating Schwann cells (S100-β+) were quantified and compared between experimental groups as described previously with the use of a square grid (Figure 3A). CM increased the number of migrating S100-β + SCs by 32% compared to the control condition (Figure 3B, C and G). Addition of neutralizing FGF-2 to the CM attenuated the increase by 48% (Figure 3, D and G). Addition of rhFGF-2 to the culture medium had an effect similar to the CM, and the neutralization of FGF-2 blocked this positive effect (Figure 3E, F, G). One might argue that fibroblasts and endothelial cells also migrate from the sciatic nerve explants in culture, but these cells do not express S100-β and only their cell nuclei are visible, preventing false positives in our data (Figure 3B, thin and thick arrows). Moreover, the total number of migrating cells (SC and non-SCs) is also reduced when FGF-2 is neutralized in the CM (Additional file 4: Figure S4). These results suggest that FGF-2 derived from BMMC has an important role in SC migration following nerve injury.

### Neutralization of FGF-2 reduces neurite outgrowth of DRG neurons induced by BMMC-CM

**Figure 8:**
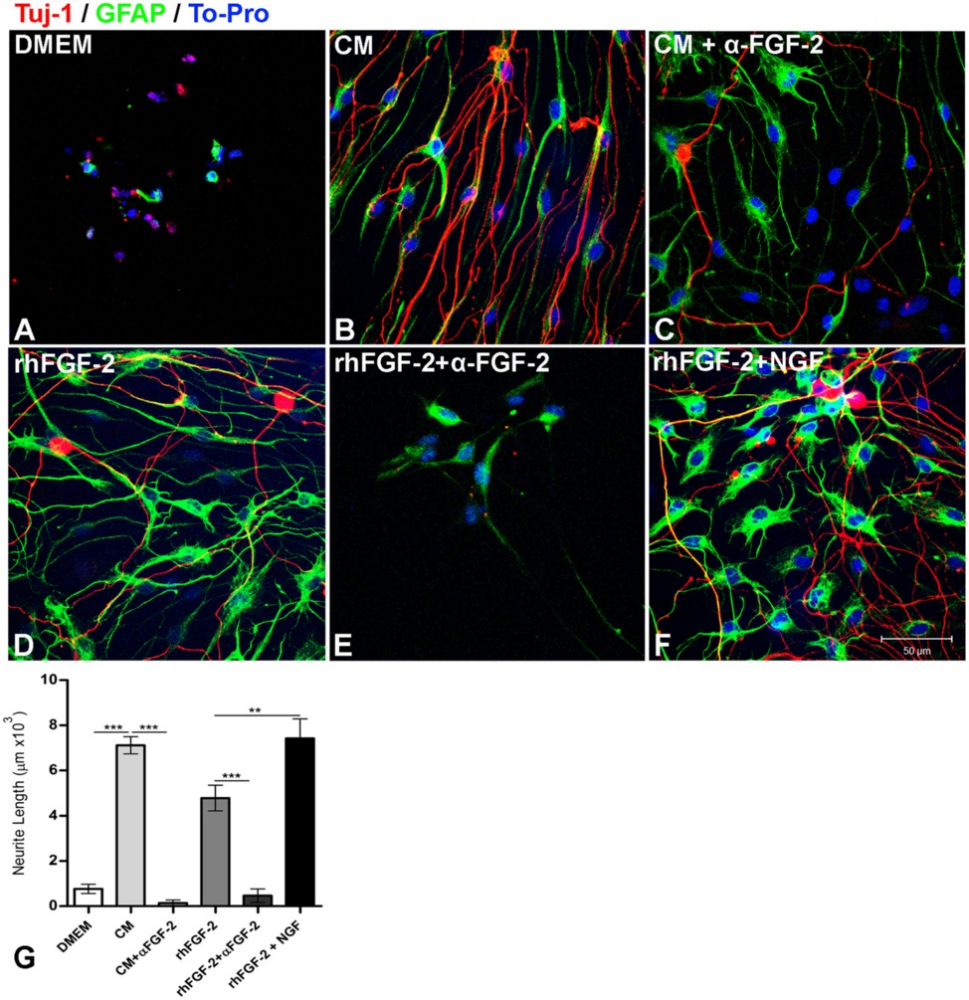
**FGF-2 secreted by BMMC stimulates neuritogenesis of DRG neurons co-cultured with SC. A**-**F**: Optical sections obtained by confocal microscopy of E16 rat embryo DRG sensory neurons (Tuj-1, red) and SCs (GFAP, green) incubated for 48 h with DMEM F-12 (control medium, **A**), bone marrow conditioned medium (CM, **B**), CM + neutralizing FGF-2 antibody (**C**), rhFGF-2 added to the control medium (**D**), rhFGF-2 + neutralizing FGF-2 antibody (**E**) or rhFGF-2 + rhNGF (**F**). Both cell types derived from cultured DRG explants of E16 rat embryos. Cell nuclei were counterstained with TO-PRO. All culture media were serum-free. rh: recombinant human protein. **G**: Quantification of the average neurite length per neuron under the experimental conditions described above. Statistics: *p* < 0.001. Scale bars: A-F = 50 μm.

It is well-known that FGF-2 binds to FGFR-3 in DRG neurons and that activation of this receptor promotes neuronal survival and axonal growth [10]. To confirm the presence of FGF-2 in the CM, dissociated DRG neurons were cultured under the same experimental conditions as described above for SCs. DRG neurons incubated with CM (Figure 8B, G) exhibited extensive neurite outgrowth 48 h after plating (4-fold increase) compared to the control group (Figure 8A, G). The neutralization of FGF-2 reduced neurite outgrowth to basal levels (Figure 8C), while addition of rhFGF-2 (20 ng/mL) to the medium also increased neurite outgrowth, but not to the levels elicited by CM (Figure 8D, G). Addition of both FGF-2 and nerve growth factor (NGF, 20 ng/mL) to control medium induced a strong neurite outgrowth similar to the neurons incubated with CM (Figure 8F-G). Altogether, our data provide strong evidence that FGF-2 is secreted by BMMC. Moreover, this FGF-2 promotes glial survival and proliferation and neurite outgrowth of rat DRG neurons.

## Discussion

Trophic activity derived from BMMC is a fundamental issue in the stem cell field and the identication of neuro and glial factors expressed in small amounts might be useful to therapies on regeneration. Several mechanisms have been suggested to explain how BMMC contribute to improve cell therapy following nerve injury [1,5,15,19,20]. Initial observations suggested that bone marrow multipotent stem cells could differentiate into any neural cell type depending on the environment conditions [21-24]. However, the number of presumed differentiated cells could not explain some of the improvements observed in the experimental rodent models. Recently, several molecules secreted from BMMC including nerve growth factor (NGF), brain derived neurotrophic factor (BDNF), cilliary neurotrophic factor (CNTF), vascular endothelial growth factor (VEGF), transforming growth factor β1 (TGF-β1) and interleukin-6 (IL-6), were identified as possible candidates that support cell therapy [25-28]. These soluble factors activate specific receptors to induce survival, growth, proliferation, migration and differentiation of specific cell types such as neural precursors, neurons, glial cells and vascular cells [1,3,29-31].

In our previous work, we demonstrated that bone marrow-derived soluble factors contribute to sciatic nerve regeneration by promoting neuronal survival, axonal growth and glial cell proliferation [5], including SCs from the proximal stump of the transected nerve, satellite cells of DRG and astrocytes surrounding motoneurons of the LSC. We also characterized NGF as a trophic factor produced and secreted by BMC because its neutralization dramatically reduced the neuritogenesis of DRG neurons induced by BMC-CM. FGF-2 has also been well characterized as a mitogen for glial cells during the development and regeneration of the central nervous system (CNS) and PNS [10]. Moreover, the survival of peripheral glial cells and their progression through the cell cycle occurs through the activation of FGFR 1/2.

It is widely known that activation of PI-3 kinase (PI3K)/Akt leads to cell survival, whereas mitogen-activated protein kinase (MAPK) activation induces cell proliferation [10]. In addition, DRG neurons express FGFR-3, which is activated by FGF-2, promoting neuronal survival and neuritogenesis by PI3K and MAPK signaling pathways respectively, *in vitro* and *in vivo*[10,32]. Here, we demonstrate that BMC-CM induces survival and proliferation of SCs and neuritogenesis of DRG neurons (Figure 6). The neutralization of FGF-2 reduced the positive effects previously observed in both cell types. Addition of rhFGF-2 to culture medium promoted similar effects, but these effects were not as strong as those observed in SCs or neurons incubated with CM. This suggests that other possible unidentified soluble factor(s) secreted by BMC might also affect cell survival, proliferation and neuritogenesis (Figure 7F-H; Figure 8). Indeed, this hypothesis is supported by the fact that NGF is one of these factors [5]. Another point observed was that when FGF-2 activity was blocked in the CM the neurite growth was almost blocked as well (Figure 8G). Since NGF was found in the CM, would be expected to observe strong neurite growth after FGF-2 neutralization. However, it was reported that when the activity of FGF receptor is blocked, MAP kinase signaling pathway triggered by NGF is severely committed [33].

Here, we provide strong evidence that BMC express and release FGF-2. Injection of BMC inside the hollow tube containing the proximal and distal nerve stumps supplies additional FGF-2 during the early period of regeneration. This might increase the SC proliferation rate. However, increased proliferation of satellite cells surrounding sensory DRG neurons and astrocytes close to the motoneurons in LSC was also observed (Figures 2, 5, 6).

It is well established that soluble molecules such as trophic factors (e.g., NGF, BDNF, NT-3 and FGF-2) are transported along axons in both anterograde and retrograde directions [3,34,35]. Therefore, it is tempting to speculate that FGF-2 secreted by BMC at the lesion site could be transported to DRGs and LSC via axonal transport. Moreover, absence of electrical signaling leads to apoptosis of injured DRG neurons and shrunken motoneurons in the CNS [36]. BMC treatment induces FGF-2 overexpression in both DRG (Figure 5) and LSC (Figure 6). It is possible that glial proliferation is stimulated by FGF-2 from multiple sources such as BMMC that activates neuronal cell bodies by axonal transport and by autocrine release by neurons and glial cells. Neutralization of FGF-2 concomitantly with BMC treatment at the lesion site reduced FGF-2 expression in DRG and LSC tissue (data not shown). This observation supports the hypothesis that bone marrow-derived FGF-2 stimulates local FGF-2 expression by DRG and LSC, as well as the axonal transport of this factor to the neuronal cell bodies. BMC treatment also leads to an increased in the proliferation of satellite cells and astrocytes. Both cell types might be ensheathing and contacting sensory and motor neurons with transected axons. Because glial cells provide trophic support to neurons [37], an increased number of these cells would lead to an increase in trophic factors. This explanation can be supported by our previous observation that BMC treatment enhances glial cell proliferation and neuronal survival [5]. Interestingly, increased levels of FGF-2 with bone marrow cells therapy have also been reported in regenerating tissue in a model of optic nerve crush lesions [15].

FGF-2 is a mitogenic factor for SCs and can also induce migration of glial cells *in vitro* which includes SC. Data from *in vitro* sciatic nerve explants showed that FGF-2 neutralizing antibody added to the CM reduces SC migration (Figure 3). However, the total number of migrating cells significantly decreased under this experimental condition. It is known that endothelial cells and fibroblasts also migrate from the explants, but the numbers of these migrating cells were not altered (data not shown). Therefore, we suggest that the effect of soluble FGF-2 in the CM is mainly on SCs in migratory experiments. Based on these results, we confirm that FGF-2 neutralization reduces the migratory process *in vitro* and we conclude that FGF-2 secreted by BMC regulates glial cell proliferation and migration. These phenomena are important because the peripheral glial cells provide a suitable environment for neuronal survival and axonal regeneration after nerve lesion. Moreover, an increase in the number of SC as well as in their migration could results in an increase in nerve regeneration and functional improvements.

Finally, we show that the presence of regenerating nerve tissue increases FGF-2 mRNA transcript levels using a model of co-cultured sciatic nerve pieces and MSC. In the absence of nerve fragments, BMMC or MSC had weak or undetectable signals for FGF-2 mRNA by RT-PCR (Figure 7I). These observations suggest an up-regulation of FGF-2 expression by SCs and MSC since both cell types stimulate each other to overexpress this trophic factor (Figures 2 and 7). We demonstrated that FGF-2 is increased at the transcript level when pieces of injured sciatic nerve were added to the culture of mesenchymal cells. However, the amount FGF-2 transcripts is reduced in BMMC when the same samples of nerve were added to the culture. Since mesenchymal cells represent around 0,01% of bone marrow mononuclear cells [18], we suggest that these cells are the main source of FGF-2. In autocrine or paracrine loops involving trophic factors, it is well understood that the first step toward overexpression of a factor is to raise the amount of the receptor in the plasma membrane [33]. The presence of more FGFRs at the cell surface enhances the response to FGF-2. As described above for SCs and MSCs, the same principle might apply to the interaction of DRG satellite cells and LSC astrocytes with MSCs. Further studies are necessary to confirm this hypothesis.

In conclusion, this work clearly demonstrates that bone marrow-derived FGF-2 contributes to peripheral nerve regeneration by stimulating glial cell survival and proliferation. Together with recent reports, this work supports the hypothesis that different bone marrow-derived molecules are working together during peripheral nerve regeneration leading to reduction of neuronal death and increasing axonal growth. Consequently, these events contribute for regeneration of nerve tissue and functional recovery of the injured PNS.

## Competing interests

The authors declare that they have no competing interests.

## Authors’ contributions

VTRR contributed to the general administration, generation of *in vivo* experimental model, histology procedures, cell culturing, fluorescence imaging, interpretation of experimental results, and development and writing of the manuscript. ACR performed cell culturing, generation of *in vivo* experimental model, histology procedures, fluorescent imaging, statistical analysis and manuscript writing. RMRL performed Schwann cell culture, biochemistry assays of western blotting and quantitative analysis by densitometry. RAMR establishing culture of neuronal and glial cell from DRG samples and writing of manuscript. RMO writing of manuscript and direction of the project. All authors read and approved the manuscript.

## Supplementary Material

Additional file 1 ** Figure S1.** Optical slice of a longitudinal section of sciatic nerve taken by confocal microscopy 10 days after injury and double immunolabeled for Erb-B2 (green) and FGF-2 (red). Colocalization of both markers is represented by yellow dots (arrows). Scale bar = 50 μm. Click here for file

Additional file 2 **Figure S2.** Quantitative analysis of GFAP reactivity in the DL LSC after sciatic nerve transection and treatment with PBS, BMMC, or BMMC + neutralizing FGF-2 antibody. Ipsi and contra-lateral sides to the nerve lesion were quantified. Statistics: *p* < 0.001 ANOVA. Click here for file

Additional file 3 ** Figure S3.** Quantitative analysis of the number of KI-67 + SCs cultured at high density (5.00 x 104) and incubated with DMEM F-12 (control medium), BMMC-CM, BMMC-CM + neutralizing FGF-2 antibody, rhFGF-2 added to the control medium or rhFGF-2 + neutralizing FGF-2 added to the standard medium. Statistics: *p* < 0.0001 ANOVA. Click here for file

Additional file 4 ** Figure S4.** Quantitative analysis of the number of migrated cells from explants of crushed sciatic nerve of adult rats under the same conditions as described in Figure 6F**.** Statistics: *p* < 0.001 ANOVA. Click here for file
