# Supplementary figures and images for: Bone marrow-derived fibroblast growth factor-2 induces glial cell proliferation in the regenerating peripheral nervous system
Source: Mol Neurodegener. 2012 Jul 13;7:34. doi: 10.1186/1750-1326-7-34 (PMC3503565; doi:10.1186/1750-1326-7-34)

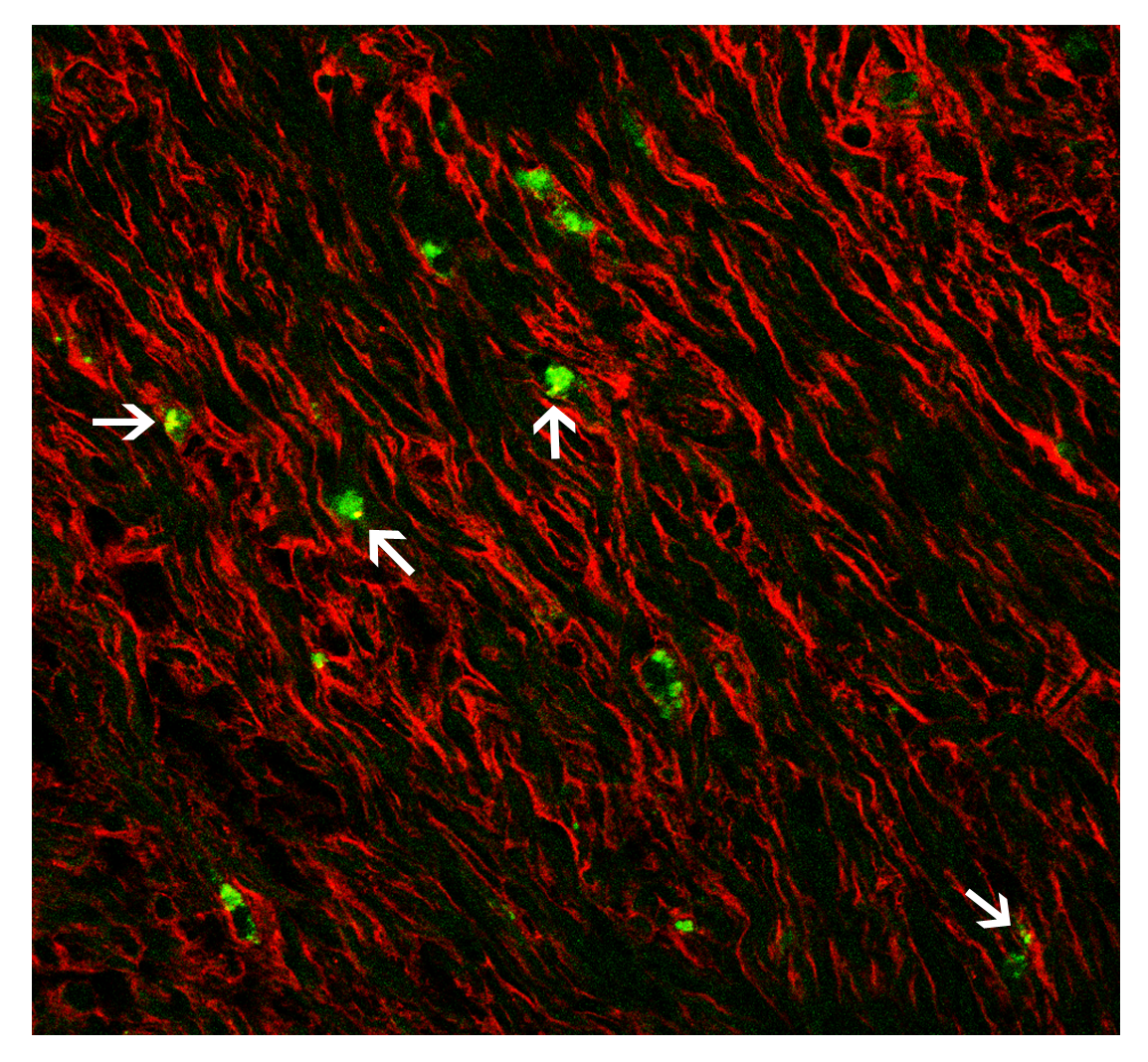

Supplement: Additional file 1 — Figure S1. Optical slice of a longitudinal section of sciatic nerve taken by confocal microscopy 10 days after injury and double immunolabeled for Erb-B2 (green) and FGF-2 (red). Colocalization of both markers is represented by yellow dots (arrows). Scale bar = 50 μm. [file 1750-1326-7-34-S1.tiff]

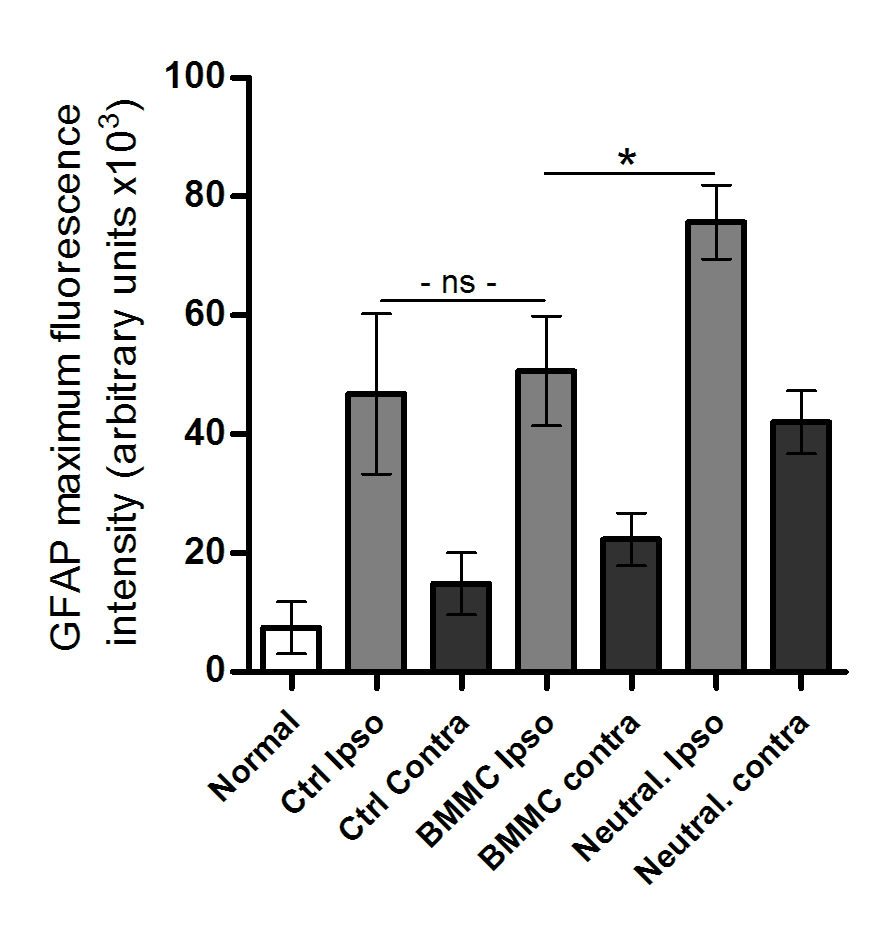

Supplement: Additional file 2 — Figure S2. Quantitative analysis of GFAP reactivity in the DL LSC after sciatic nerve transection and treatment with PBS, BMMC, or BMMC + neutralizing FGF-2 antibody. Ipsi and contra-lateral sides to the nerve lesion were quantified. Statistics: p < 0.001 ANOVA. [file 1750-1326-7-34-S2.tiff]

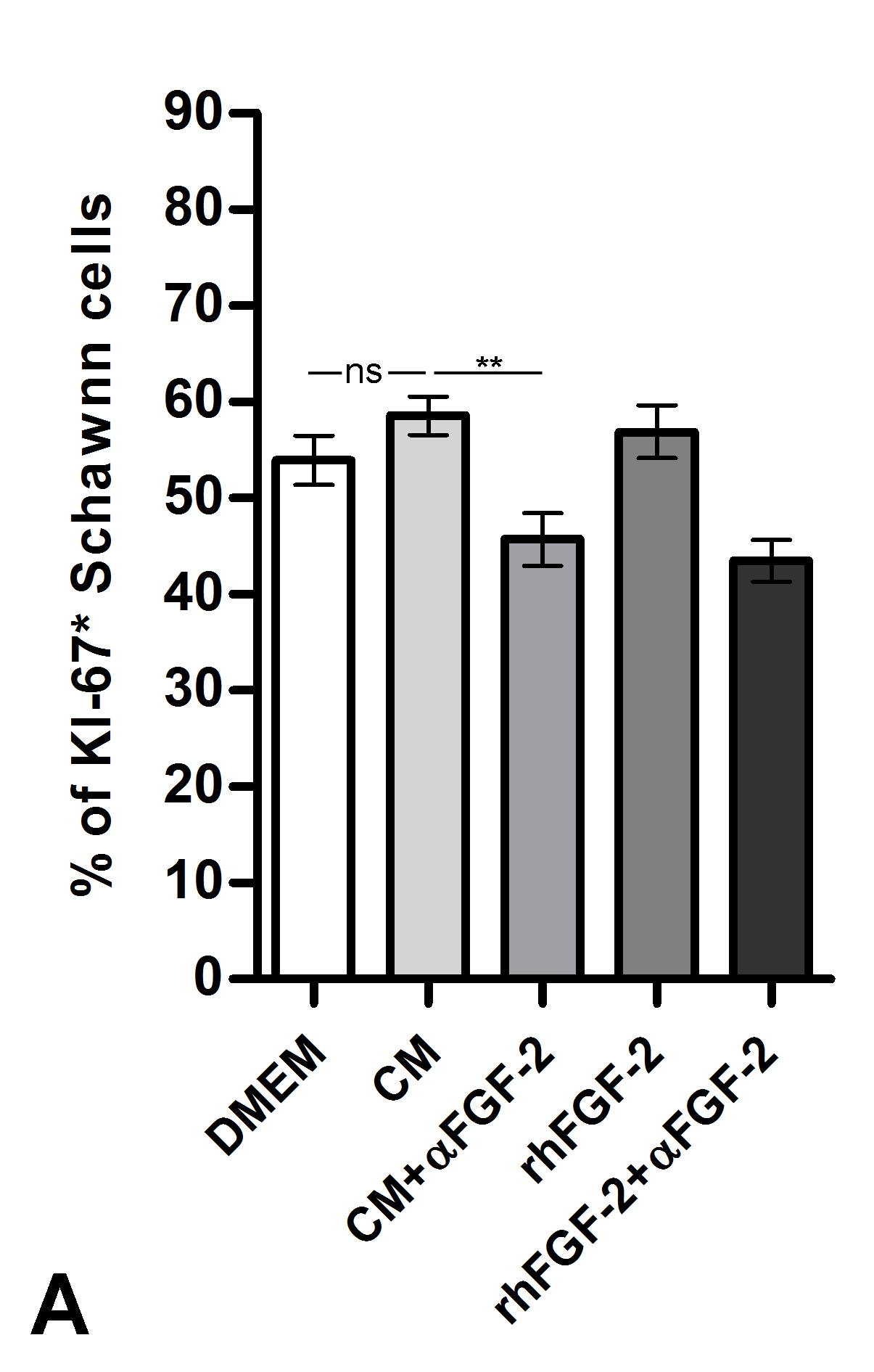

Supplement: Additional file 3 — Figure S3. Quantitative analysis of the number of KI-67 + SCs cultured at high density (5.00 x 10^4^) and incubated with DMEM F-12 (control medium), BMMC-CM, BMMC-CM + neutralizing FGF-2 antibody, rhFGF-2 added to the control medium or rhFGF-2 + neutralizing FGF-2 added to the standard medium. Statistics: p < 0.0001 ANOVA. [file 1750-1326-7-34-S3.tiff]

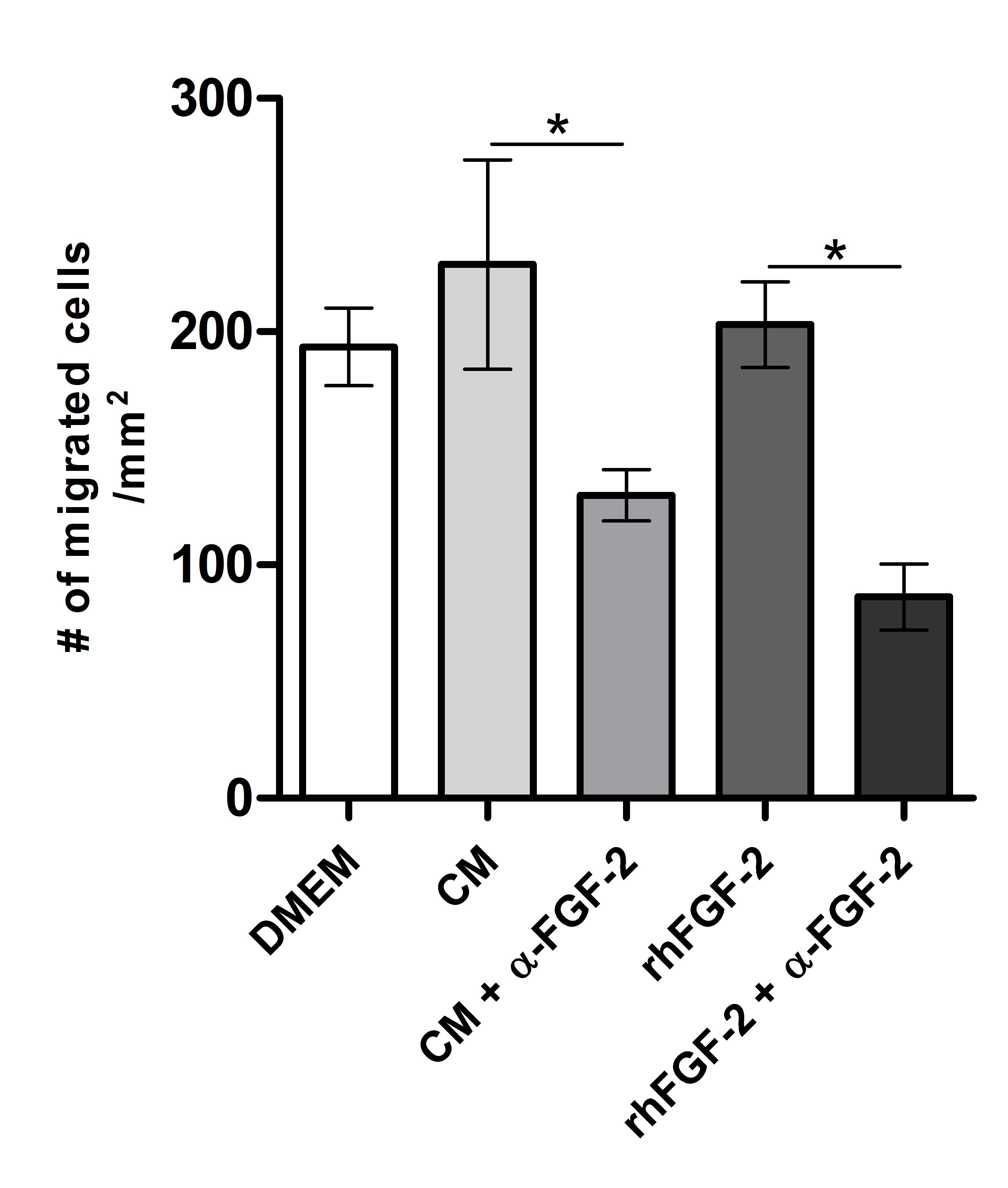

Supplement: Additional file 4 — Figure S4. Quantitative analysis of the number of migrated cells from explants of crushed sciatic nerve of adult rats under the same conditions as described in Figure 6F. Statistics: p < 0.001 ANOVA. [file 1750-1326-7-34-S4.tiff]
